# Spermidine induces cytoprotective autophagy of female germline stem cells in vitro and ameliorates aging caused by oxidative stress through upregulated sequestosome-1/p62 expression

**DOI:** 10.1186/s13578-021-00614-4

**Published:** 2021-06-07

**Authors:** Xiaoyan Yuan, Geng. G. Tian, Xiuying Pei, Xiaopeng Hu, Ji Wu

**Affiliations:** 1 Key Laboratory of Fertility Preservation and Maintenance of Ministry of Education, Ningxia Medical University, Yinchuan, China; 2 Key Laboratory for the Genetics of Developmental & Neuropsychiatric Disorders (Ministry of Education), Bio-X Institutes, Shanghai Jiao Tong University, Shanghai, China; 3 Department of Emergency Medicine, Gongli Hospital, Pudong New Area, Shanghai, China

**Keywords:** Spermidine, Autophagy, Female germline stem cells, Anti-aging, Anti- oxidative stress, p62

## Abstract

**Background:**

Autophagy is required for oogenesis and plays a critical role in response to aging caused by oxidative stress. However, there have been no reports on regulation of cytoprotective autophagy in female germline stem cells (FGSCs) in response to aging caused by oxidative stress.

**Results:**

We found that Spermidine (SPD) significantly increased protein expression of autophagy markers microtubule-associated protein 1 light chain 3 beta-II (MAP1LC3B-II/LC3B-II) and sequestosome-1/p62 (SQSTM1/p62), and evoked autophagic flux in FGSCs. Moreover, SPD increased the number and viability of FGSCs in vitro. Further, we found that SPD significantly reduced basal or hydrogen peroxide (H_2_O_2_)-induced up-regulated protein expression of the aging markers, cyclin dependent kinase inhibitor 2A (p16/CDKN2A) and tumor protein 53 (p53). After knockdown of p62 in FGSCs, p16 protein levels were significant higher compared with controls. However, protein p16 levels were not significantly changed in p62 knockdown FGSCs with SPD treatment compared with without SPD. Moreover, SPD significantly changed the expression of autophagy-related genes and pathways in FGSCs, as shown by bioinformatics analysis of RNA sequencing data. Additionally, SPD significantly inhibited AKT/mTOR phosphorylation.

**Conclusions:**

SPD induces cytoprotective autophagy in FGSCs in vitro and ameliorates cellular senescence of FGSCs induced by H_2_O_2_. Furthermore, SPD can ameliorate cellular senescence of FGSCs through p62. SPD might induce autophagy in FGSCs via the PI3K/Akt pathway. Our findings could be helpful for delaying aging of female germ cells due to oxidative stress and preserving female fertility.

**Supplementary Information:**

The online version contains supplementary material available at 10.1186/s13578-021-00614-4.

## Introduction

Although hyper-activation of autophagy is believed to cause autophagy-dependent cell death, autophagy primarily protects against cellular insults, nutrient starvation, or oxidative stress. Autophagy, which is an evolutionarily conserved process of organisms, ensures cellular homeostasis and proteostasis, and is directly involved in degradation of damaged, potentially toxic organelles and harmful protein aggregates. This process removes and recycles cytoplasmic material that otherwise would accumulate during aging [1]. Recent genetic evidence indicates that autophagy has a crucial role in regulating the lifespan in animal models [2]. Autophagy-related genes are required for extension of the lifespan in various long-lived mutant nematodes and promote survival in worms and flies exposed to prolonged starvation [3]. Cytoprotective autophagy is essential for cellular homeostasis [3], while a decline in activity of autophagy is a central molecular mechanism leading to aging [4, 5]. As the world population ages and aging-related diseases become more prevalent, aging mechanisms and anti-aging strategies have attracted attention worldwide in the past few years [5]. Therefore, autophagy has become one of the most targeted approaches for anti-aging. However, the relationship between the mechanism of autophagy and aging, and the effect of autophagy in regulating the lifespan remain unclear.

Female fertility declines irreversibly with aging, and advanced maternal age is mostly related to impaired quality of oocytes. A major cause of oocyte aging is increased oxidative damage by excessive reactive oxygen species (ROS), which can result in part from weakened anti-oxidative defense systems [6, 7]. Female germline stem cells (FGSCs) that are isolated from ovarian tissue of neonatal and adult mice develop into mature eggs and healthy offspring can be produced from FGSCs [8–13]. Furthermore, FGSCs have been isolated postnatally from other types of mammalian and human tissues [10, 14–18]. Successful establishment and cultivation of an FGSC line has been important for investigating development of eggs, delaying aging of ovary, and preserving female fertility. Abnormal development of FGSCs, including aging caused by oxidative damage, excessive autophagy, and apoptosis, is the source of a reduction in the number of germ cells in the ovary. Therefore, anti-aging caused by oxidative stress damage is an important aspect of delaying ovarian aging and maintaining fertility. Recent studies have shown that proliferation of FGSCs is inhibited by some small molecular compounds inducing hyper-autophagy [19–21]. However, there have been no reports on regulation of cytoprotective autophagy in FGSCs in response to aging caused by oxidative stress.

Spermidine (SPD) is a type of polyamine that is commonly found in living organisms. SPD is a natural small molecular compound synthesized by the human body, and it is also present in the daily diet and considered as a caloric restriction mimetic (CRM) [22]. For decades, research has suggested that levels of polyamines in cells decline with age and that this contributes to the pathophysiology of aging [23, 24]. Previous studies have shown that nutritional supplementation of SPD can not only restore polyamine levels in vivo, but also suppress age-induced memory impairment in *Drosophila* [24]. SPD also provides cardioprotection, reducing systemic blood pressure, delaying progression to heart failure [25], and selectively inhibiting tumor cells [26, 27]. The naturally occurring polyamine SPD has recently been shown to promote longevity across species and protect against oxidative stress [28, 29]. Notably, all of these beneficial effects have been achieved in an autophagy-dependent manner [22, 28]. However, the role of SPD in female fertility, and whether it can induce autophagy to reduce age-related oxidative stress and potentially relieve ROS-induced damage of FGSCs remain largely unknown.

In this study, we investigated the role and potential mechanisms of SPD in cytoprotective autophagy of FGSCs in response to oxidative stress, which causes FGSCs to age. We used hydrogen peroxide (H_2_O_2_) to induce aging of FGSCs and then investigated whether SPD protects FGSCs from oxidative damage induced by H_2_O_2_ in vitro. Our findings could be helpful for delaying aging of female germ cells due to oxidative stress and preserving female fertility.

## Results

### SPD induces autophagy and activates autophagic flux of FGSCs in vitro

**Fig. 1:**
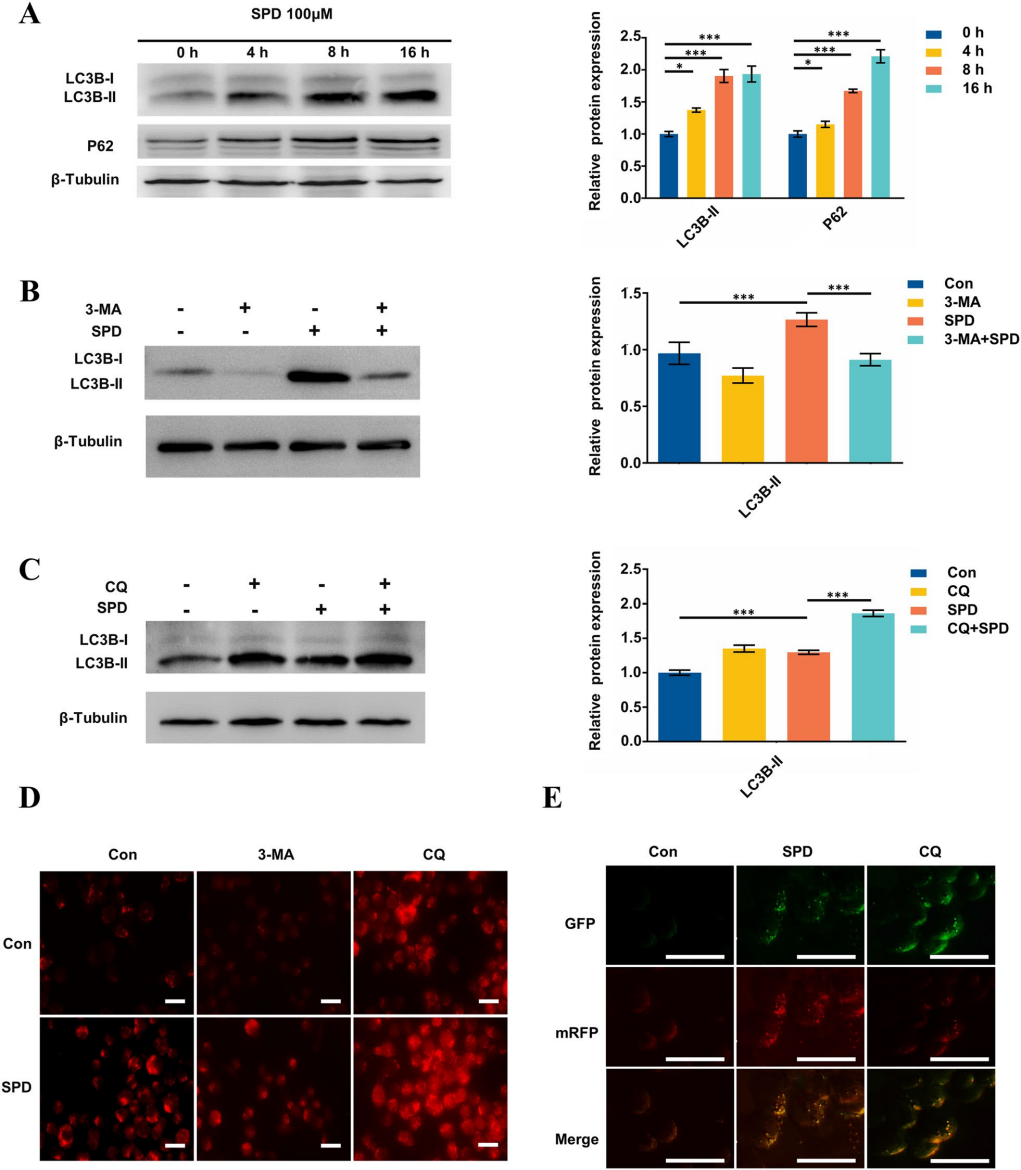
SPD induces autophagy of FGSCs and enhances autophagic flux. **A** LC3B, p62, and β-tubulin (control for loading) protein expression levels in FGSCs treated with SPD (100 μM) for different times. *P < 0.05, ***P < 0.001 compared with controls. Western blot analysis was performed to detect LC3B and β-tubulin protein levels in FGSCs pretreated with **B** 3-MA (5 mM) or **C** CQ (20 μM) for 1 h, followed by exposure to SPD (100 μM) for another 4 h. Numbers below the dots correspond to relative quantification by densitometry compared with the reference point set to 1. **D** Immunofluorescence images of LC3B puncta in FGSCs pretreated with inhibitors of autophagy (3-MA or CQ) followed by exposure to SPD (100 μM) for another 4 h. The number of LC3B puncta per cell was the highest in co-incubation of cells with SPD and CQ treatment (scale bars: 20 μm). **E** Fluorescence microscopic examination of FGSCs expressing a tandem GFP-RFP-LC3 fusion protein that were treated with SPD with or without CQ (20 μM) for 4 h (scale bars: 50 μm). *Con* control

To investigate whether SPD induces autophagy of FGSCs, we treated FGSCs with 100 μM SPD at different time points and examined protein levels of two markers of autophagy, microtubule-associated protein 1 light chain 3 beta-II (MAP1LC3B-II/LC3B-II) and sequestosome-1 (p62). We found that 100 μM SPD significantly increased LC3B-II protein expression in a time-dependent manner from 4 to 16 h (Fig. 1A). Additionally, there was a robust increase in p62 protein expression following a certain concentration of SPD (100 μM) treatment in a time-dependent manner (from 4 to 16 h) (Fig. 1A).

This upregulated LC3B-II protein expression indicated formation of autophagosomes. Additionally, increased p62 protein expression may have been associated with blocking autophagosome–lysosome fusion, which indicated that autophagic flux was inhibited. To determine whether autophagy flux is inhibited after SPD induced autophagy, three different assays were used. First, we cotreated FGSCs with SPD and inhibitors of autophagy, 3-methyladenine (3-MA) and chloroquine (CQ), which block upstream and downstream steps of autophagy process, respectively. Co-incubation of FGSCs with SPD and 3-MA (5 mM) for 4 h led to significantly lower SPD-induced LC3B-II formation compared with SPD treatment, as shown by western blot analysis (Fig. 1B). In contrast, co-incubation of cells with SPD and CQ (20 μM) led to higher conversion of LC3B-II (Fig. 1C). Second, significant accumulation of LC3B-II puncta was shown by immunostaining with co-incubation of SPD and CQ (Fig. 1D). Finally, we also generated a cell line stably expressing fusion protein mRFP-GFP-LC3 in FGSCs and mRFP is used to indicate LC3 expression level in real time. When autophagosomes fuse with lysosomes, GFP fluorescence is quenched in the acidic environment and only red fluorescence is observed, which is used to detect the level of autophagy flux. The yellow (autophagosomes) and red (autolysosomes) puncta were imaged by fluorescence microscope during CQ and SPD treatment. We observed that, during SPD treatment, red puncta were predominant, which indicated that autophagosome–lysosome fusion was not affected. In contrast, when exposed to the autophagic inhibitor CQ, the amount of LC3 punctas was much higher compared with that in untreated cells, and almost all of these punctas showed yellow fluorescence, which suggested a fusion defect (Fig. 1E). Taken together, these results indicated that SPD increased LC3BII and p62 expression to induce autophagy of FGSCs, and evoked autophagic flux in vitro.

### SPD increases the number and viability of FGSCs in vitro

**Fig. 2:**
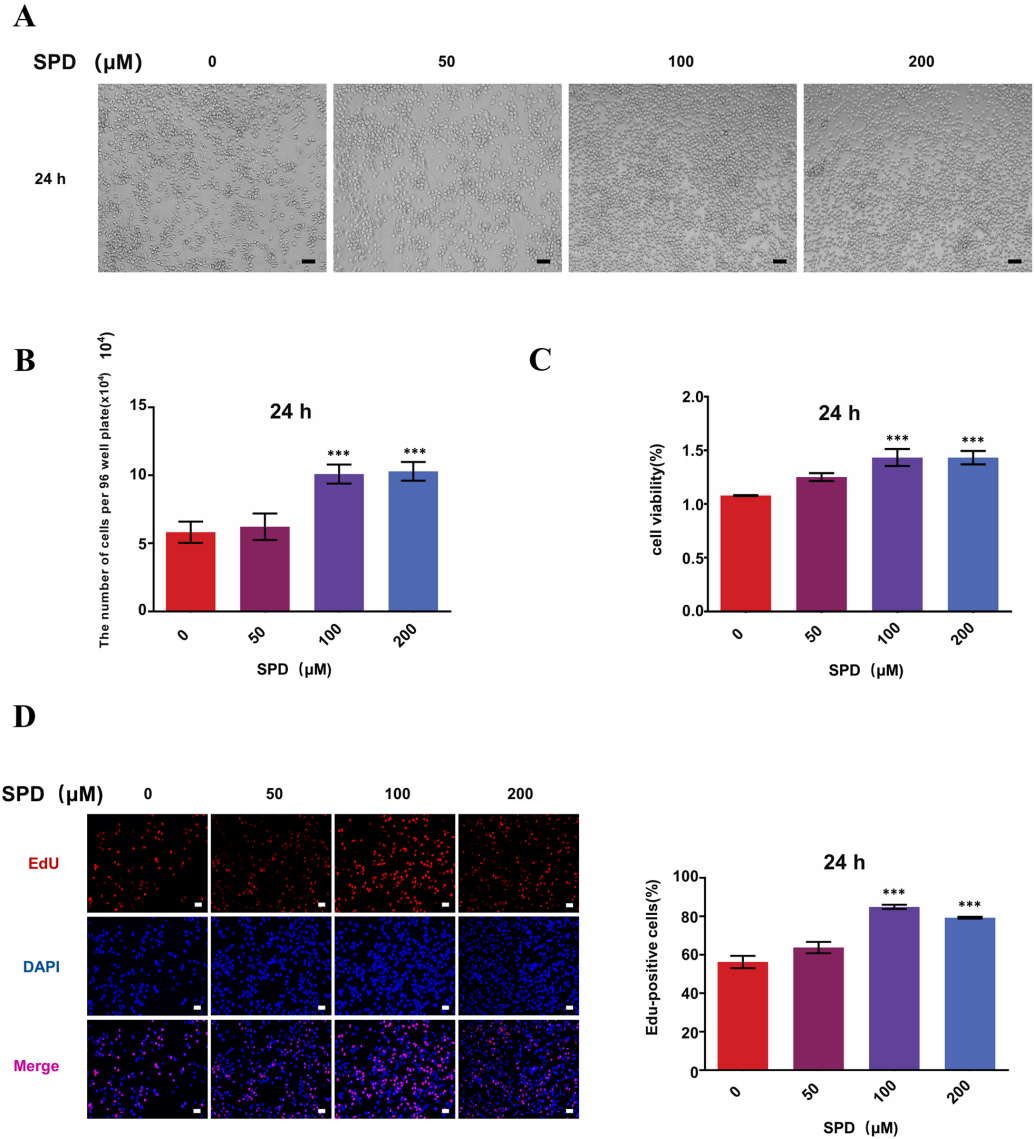
SPD enhances viability and increases the number of FGSCs. **A** Cellular morphology of FGSCs treated with various concentrations (0, 50, 100, and 200 μM) of SPD at 24 h (scale bars: 20 µm). **B** The amount of FGSCs treated with various concentrations of SPD. **C** Graphic representation of results from CCK-8 assays to determine cell viability of FGSCs treated with different concentrations of SPD for 24 h. Data points are the percentage (%; optical density at 450 nm, treated/optical density at 450 nm, untreated) relative to untreated cells at that time point. **D** Proliferation of FGSCs treated by SPD at 24 h was determined using 5-ethynyl-2ʹ-deoxyuridine staining (scale bars: 20 μm). Proliferation was significantly increased from the 100 μM SPD-treated groups. All data are expressed as the mean ± SEM of values from experiments performed at least in triplicate. ***P < 0.001 compared with controls

Autophagy can lead to cell death in response to stress, but it can also act as a protective mechanism for cell survival. Therefore, to evaluate whether SPD leads to cell death or plays a protective role in development of FGSCs in vitro, we treated the cells with differing concentrations of SPD (0, 50, 100, and 200 μM) at 24 h. The number of FGSCs was significantly higher with treatment of 100 μM SPD compared with untreated cells at 24 h (Fig. 2A, B). We then assessed the viability of FGSCs treated with different concentrations of SPD in vitro at 24 h using the cell viability assay cell counting kit 8 (CCK-8). The viability of FGSCs was significantly higher with treatment with 100 μM SPD compared with untreated cells (Fig. 2C). The proliferation rate of FGSCs was significantly higher in the 100 and 200 µM SPD-treated groups at 24 h compared with controls, as shown in 5-ethynyl-2ʹ-deoxyuridine assays (Fig. 2D). These results indicated that 100 and 200 µM SPD significantly increased the number and viability of FGSCs in vitro. Taking together, these results indicated that SPD induced cytoprotective autophagy of FGSCs in vitro.

### SPD induces cytoprotective autophagy and ameliorates aging of FGSCs caused by H_2_O_2_ in vitro

**Fig. 3:**
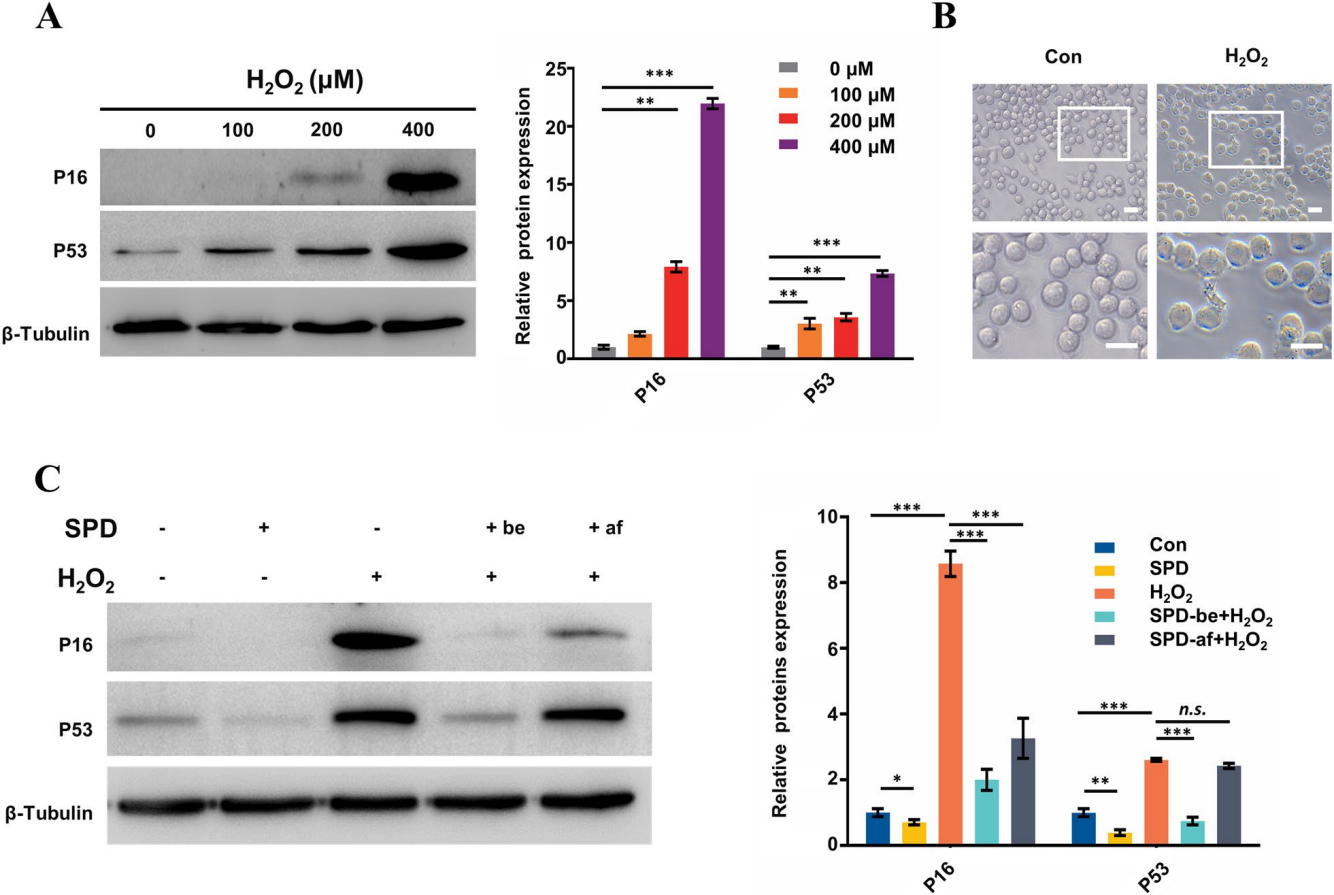
SPD ameliorates aging of FGSCs induced by H_2_O_2_. **A** Western blot analysis was performed to detect levels of the aging markers p16 and p53 in FGSCs treated with various concentrations (0, 100, 200, 400, and 800 μM) of H_2_O_2_ for 2 h. **B** Senescence-related β-galactosidase staining (blue) to detect aging of FGSCs with or without H_2_O_2_. Blue-stained cells were considered as aging of FGSCs. **C** Western blot analysis shows p16 and p53 protein levels in FGSCs with or without H_2_O_2_ (400 μM, 2 h), and with or without SPD (100 μM) treatment. Two groups were treated by SPD, including the prophylactic group with pre-exposure to SPD for 24 h before induction of H_2_O_2_ and the treatment group with exposure to SPD for 24 h after induction of H_2_O_2_. All data are expressed as the mean ± SEM of values from experiments performed at least in triplicate. *P < 0.05, **P < 0.01, and ***P < 0.001 compared with controls. *n.s.* no significance, *Con* control

Cytoprotective autophagy is associated with resistance to cellular senescence caused by oxidative stress. To test this resistant effect of SPD in FGSCs, we detected expression of cellular senescence markers in aging FGSCs induced by H_2_O_2_ following SPD treatment or no SPD treatment. First, we treated FGSCs with differing concentrations of H_2_O_2_ for 2 h. We then removed H_2_O_2_ and continued to culture cells for 24 h in fresh media to obtain the aging FGSCs model. Protein expression of p16 and p53, aging marker proteins, was upregulated with an increase in H_2_O_2_ concentrations, as shown by western blot analysis. Protein expression of p16 and p53 was significantly higher at 400 μM H_2_O_2_ compared with controls (Fig. 3A). Therefore, 400 μM H_2_O_2_ was selected for the following experiments. Additionally, FGSCs treated with H_2_O_2_ displayed cellular characteristics of senescence: positivity for senescence-related β-galactosidase staining (Fig. 3B).

We then treated FGSCs with H_2_O_2_ (400 µM), SPD (100 µM), H_2_O_2_ (400 µM) + SPD (100 µM), or control. Notably, to determine the difference in efficacy of SPD between preventive and therapeutic medication, the SPD treatment group was divided into two groups in further experiments. One group was the preventive group that was pre-exposed to SPD for 24 h before induction of H_2_O_2_ and the other was the therapeutic group that was exposed to SPD for 24 h after induction of H_2_O_2_. Protein expression levels of p16 and p53 were detected by western blot. We found that p16 and p53 levels were significantly down-regulated in the preventive group compared to the FGSCs treated with H_2_O_2_ (400 µM) alone. However, in the therapeutic group, SPD significantly down-regulated p16, but not p53 after induction of H_2_O_2_ compared to the FGSCs treated with H_2_O_2_ (400 µM) alone (Fig. 3C). These results indicated that SPD was resistant to oxidative stress causing senescence of FGSCs.

### SPD induces cytoprotective autophagy and ameliorates aging of FGSCs in vitro caused by H_2_O_2_ through upregulated p62 expression

**Fig. 4:**
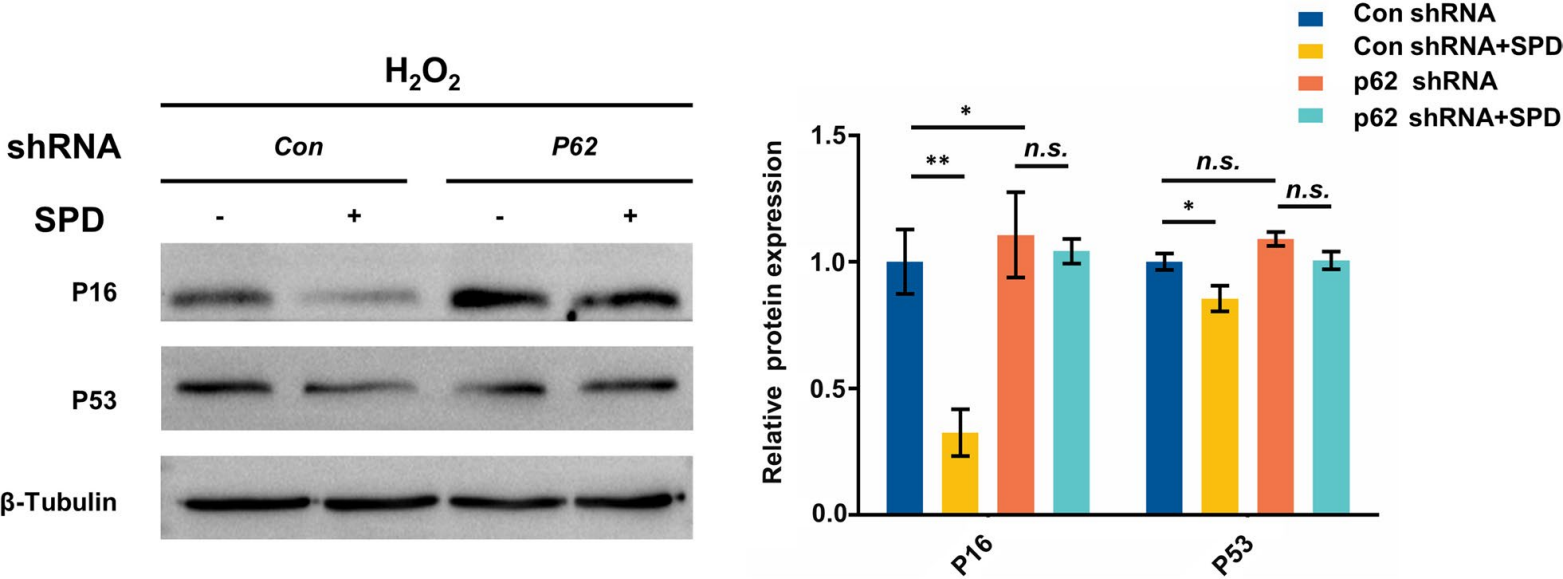
SPD improves aging of FGSC depending on upregulation of p62. Protein expression of p16 and p53 in p62-kd and control-kd FGSCs with or without SPD treatment after induction by H_2_O_2_. Data are representative of at least three independent experiments. *P < 0.05, * P < 0.01, and ***P < 0.001 compared with controls. *n.s.* no significance, *kd* knockdown, *Con* control

Our above-mentioned results indicated that p62 protein expression was upregulated in FGSCs by SPD. A recent study showed that overexpression of p62 not only induced autophagy, but also extended the lifespan, and improve the fitness of mutants with defects in proteostasis in an autophagy-dependent manner [30]. Therefore, we further investigated whether p62 is involved in the mechanism of SPD in anti-aging of FGSCs damaged by oxidative stress (H_2_O_2_). In this experiment, p62 short hairpin RNA (shRNA) was transfected into FGSCs to inhibit p62 protein expression in the presence of SPD, and p16 and p53 protein levels were examined by western blotting. We found that p16 protein expression were significantly higher in p62 knockdown FGSCs than in control knockdown cells. SPD led to significantly lower p16 and p53 protein expression in control knockdown cells compared with without SPD treatment. However, p16 and p53 protein levels were not significantly changed in p62 knockdown FGSCs with SPD treatment compared with without SPD treatment (Fig. 4A, B). These results indicated that SPD ameliorated cellular senescence of FGSCs damaged by H_2_O_2_ oxidative stress and decreased p16 expression through upregulation of p62 expression. Expression of p62 plays a critical role in autophagy of FGSCs mediated by SPD.

### SPD changes expression of autophagy-associated genes and pathways in FGSCs

**Fig. 5:**
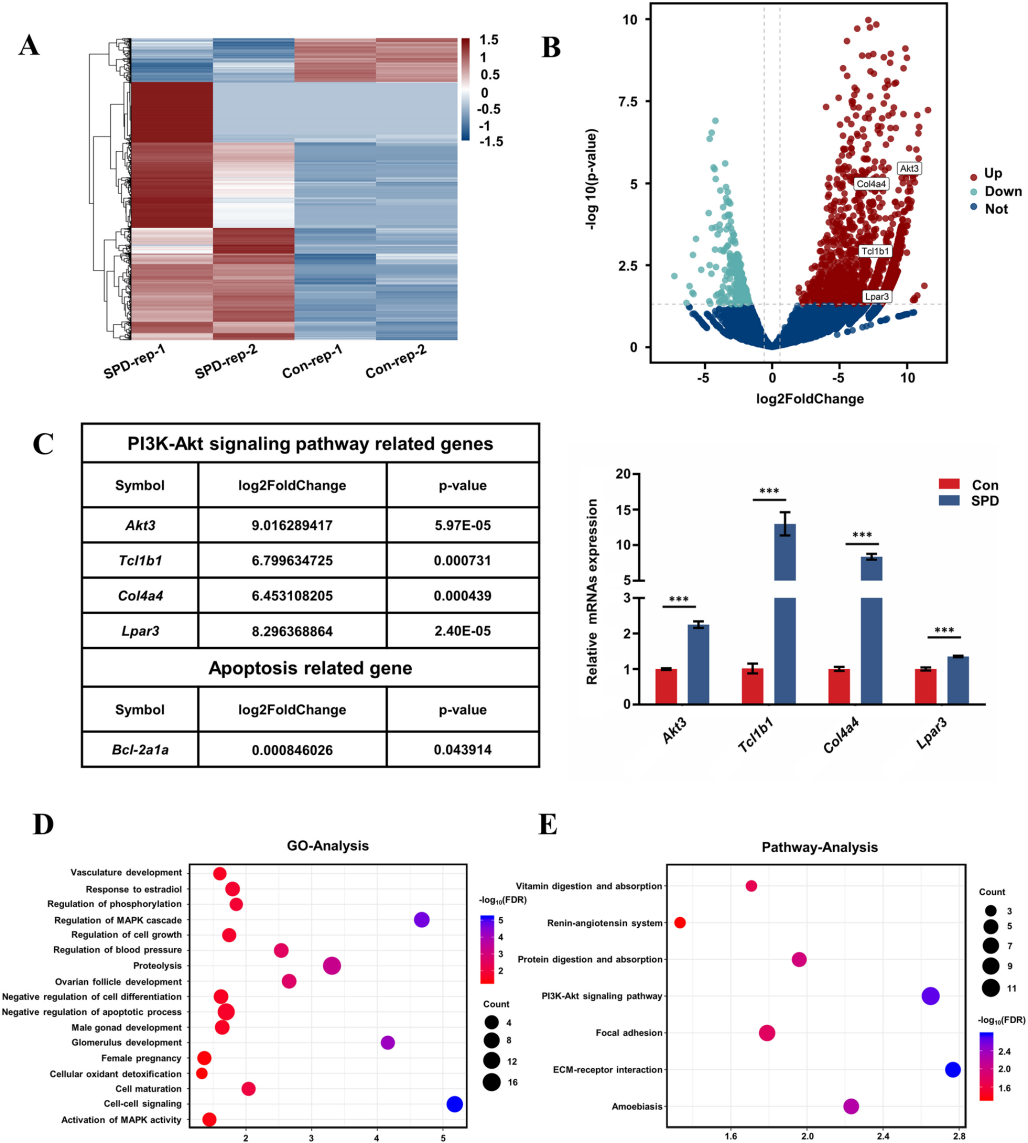
SPD changes expression of autophagy-related genes and pathways in FGSCs. **A** Hierarchical clustering shows differentially expressed mRNA patterns between the control and SPD-treated groups. **B** Volcano plot of differentially expressed mRNAs. **C** Differentially expressed mRNAs are related to genes involved in the PI3K/AKT pathway. The qRT-PCR results are consistent with changes in the transcriptome profile (P < 0.05). ***P < 0.001 compared with controls. Note that one of the differentially expressed mRNAs is *Bcl-2*, which can inhibit cell death. **D** GO analysis of RNA-seq data. The top 17 significantly enriched biological processes are shown. **E** KEGG pathway analysis of RNA-seq data. The top seven enriched pathways are shown. *Con* control

To identify the mechanisms underlying SPD-induced cytoprotective autophagy in FGSCs, we performed RNA sequencing (RNA-seq) analysis of FGSCs after treatment with 100 µM SPD for 4 h. The RNA sequencing (RNA-seq) data were analyzed by bioinformatics methods. We required a Q-score > 10 (error rate < 10%) for each replicate. Moreover, the correlation coefficient of each replicated sample showed high consistency (Additional file 1: Fig. S1). We then analyzed the RNA-seq data after quality screening. After applying a stringent filtering approach that compared control and SPD-treated groups (adjusted P < 0.05; fold change > 1.5), we identified 2313 upregulated and 392 downregulated mRNAs. A heat map (Fig. 5A) and a volcano plot (Fig. 5B) of the differentially expressed mRNAs were generated by R version 3.5.3 (https://www.r-project.org). Hierarchical clustering of differentially expressed mRNAs showed a clear distinction between the control and SPD-treated groups. Among the significantly differentially expressed mRNAs, 11 mRNAs were related to the phosphatidylinositol 3ʹ-kinase (PI3K)/AKT signaling pathway, which is a classical autophagy pathway, such as *AKT3, Tcl1b1, and Col4a4.* Expression of these mRNAs was verified by quantitative reverse transcription-polymerase chain reaction (qRT-PCR), and the results of qRT-PCR were consistent with RNA-seq data (Fig. 5C). We found that B cell leukemia/lymphoma 2 (*Bcl2*) mRNA was also upregulated with SPD. The *Bcl-2* gene is a type of oncogene, which can significantly inhibit apoptosis of cells. In our experiments, SPD significantly increased the number of FGSCs (Fig. 2A, B) and promoted cellular proliferation in vitro (Fig. 2D). The results of RNA-seq were consistent with this finding.

Gene ontology (GO) analysis was used to identify the functional categories of these differentially expressed mRNAs. A total of 17 GO terms in the category of biological processes were significantly enriched at a false discovery rate threshold of < 0.05. There were some biological processes related to cellular development, such as development of ovarian follicles, regulation of cell growth, and negative regulation of the apoptotic process (Fig. 5D). These results are consistent with the in vitro data, which showed that SPD increased the number, viability, and proliferation of FGSCs. Kyoto Encyclopedia of Genes and Genomes (KEGG) pathway analysis showed that the top seven enriched pathways were the PI3K/AKT signaling pathway, focal adhesion, amoebiasis, extracellular matrix-receptor (ECM-receptor) interaction, protein digestion and absorption, vitamin digestion and absorption, etc. (Fig. 5E). The PI3K/AKT pathway is one of the most important signaling pathways of autophagy. RNA-seq showed that differentially expressed mRNAs and enriched pathways were closely related to autophagy. These results indicated that SPD changed the expression of autophagy-related genes and signaling pathways in FGSCs.

### SPD activates autophagy via inhibition of AKT/mTOR phosphorylation

**Fig. 6:**
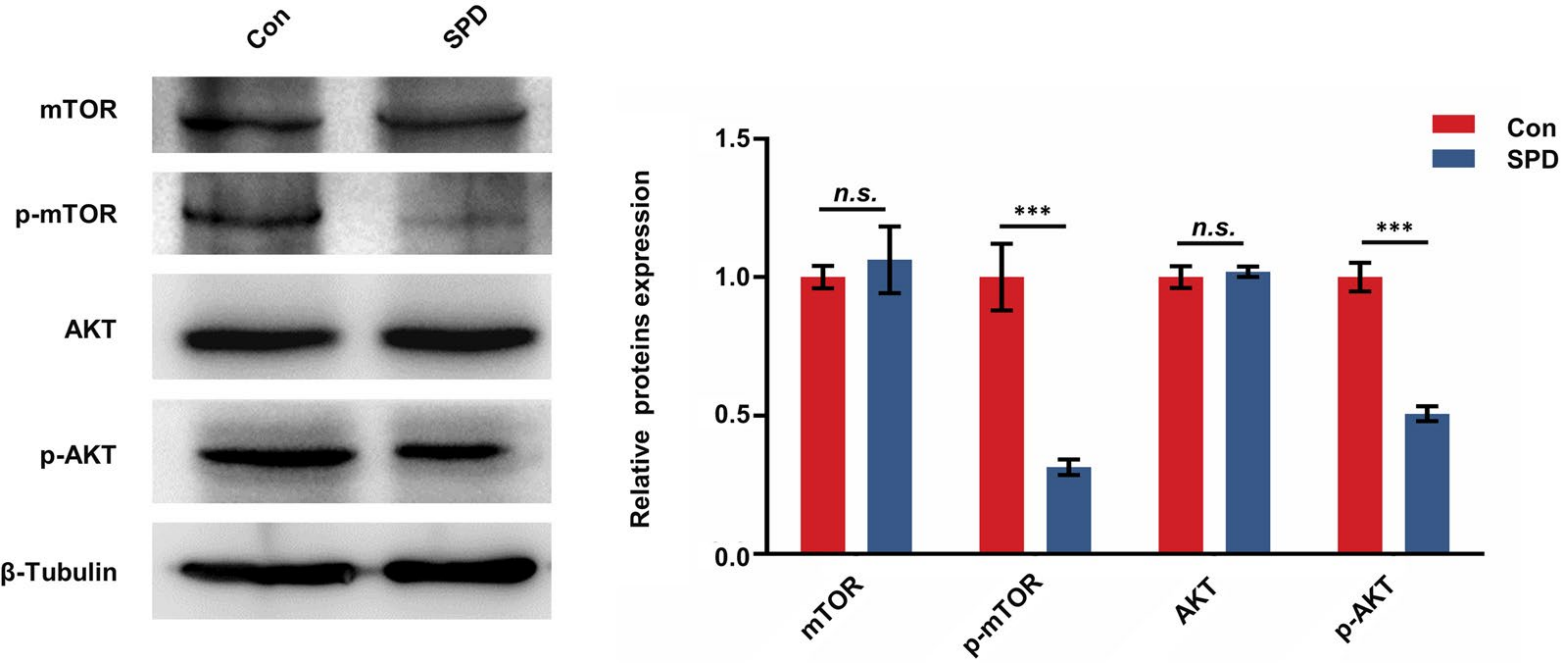
SPD induces autophagy through the AKT/mTOR signaling pathway in FGSCs. Western blot analysis of AKT, p-AKT, mTOR, and p-mTOR protein levels in FGSCs with or without SPD treatment for 4 h. ***P < 0.001 compared with controls. *n.s.* no significance, *Con* control

Based on bioinformatics analysis of RNA-seq data, SPD activated autophagy maybe via inhibition of AKT/mTOR phosphorylation. Therefore, to examine the effect of SPD on the PI3K/AKT pathway in FGSCs, we compared protein expression levels of AKT, phosphorylated AKT (p-AKT), mTOR, and phosphorylated mTOR (p-mTOR) in FGSCs after with or without SPD treatment. Protein p-AKT and p-mTOR levels were significantly lower in FGSCs treated with SPD for 4 h compared with controls (Fig. 6). These results suggested that treatment with SPD restrained the PI3K/AKT/mTOR pathway by inhibiting phosphorylation of AKT and mTOR, and thus activated autophagy.

## Discussion

SPD is a natural inducer of autophagy with no toxic side effects. SPD improves memory loss and cardiovascular and nervous system diseases caused by aging, regulates blood pressure (related biological processes was enriched by GO analysis of RNA sequencing data in this study), and it also has a selective inhibitory effect on tumor cells [24–27, 31]. However, whether SPD plays a role in oogenesis and development of germline stem cells is unknown. The viability of FGSCs plays a critical role in maintenance of normal oogenesis, which is important for determining a young state of the body. Therefore, maintaining the vitality of germline stem cells can not only increase fertility, but also play an important role in delaying the aging process. SPD is present in cells of the body and in daily human nutrition, and it has many functions for maintaining health and preventing disease [22]. Therefore, SPD has been receiving an increasing amount of attention from scientists. Our study showed that SPD not only activated autophagy of FGSCs cultured in vitro, but also enhanced autophagy flux of FGSCs cultured in vitro. We also found an increased number and enhanced viability of FGSCs treated with SPD.

With aging of the world’s population, methods of combating disease and death caused by aging have become a major strategic issue. Aging refers to the phenomenon that the morphology, structure and physiological function of organisms decline with age. Because the lifespan of cells cultured in vitro is proportional to longevity of the body, senescence of cells is often used to reflect the aging process of corresponding tissue [32]. At the molecular level, changes in cellular senescence generally refer to shortening of telomere length, weakening of proliferation and viability of cells, an increase in cell senescence-related proteins, enhancement of senescence-related β-galactosidase activity, and weakening of DNA damage repair ability. Although there have been many studies on the mechanism of aging and anti-aging strategies [5, 33–35], controlling, delaying, or reversing aging is still not possible. In our study, when aging of FGSCs was induced by H_2_O_2_ in vitro, the FGSCs were treated with SPD and divided into the prophylactic and treatment groups. We found that SPD had an obvious anti-aging effect on cellular senescence of FGSCs caused by oxidative stress injury. Additionally, the effect of prophylactic administration was much better than that of therapeutic administration. This finding suggests that the protective effect of SPD on FGSCs may mainly be due to its effect of reducing oxidative stress injury, which causes aging of cells.

In the experiment of inducing autophagy of FGSCs by SPD, we found an uncommon phenomenon in that SPD upregulated expression of the autophagic receptor p62 in a time-dependent manner. As a selective autophagic receptor, p62 is degraded by the autophagy–lysosome pathway through interaction between LC3 and the LC3 recognition sequence (LRS), which is a special region on p62 [36, 37]. Concurrently, p62 is an important guarantee for the activation of autophagic flux during downstream of autophagic process owing to its ability to bind the ubiquitinated autophagic substrates through the UBA domain, and the ability to interact with LC3 through the LIR domain [36]. However, our study showed that accumulation of p62 was not caused by inhibition of clearance of autophagosomes, and SPD promoted autophagy flux as well as up-regulating p62. SPD can up-regulate the expression of p62, which has also been shown in previous research on SPD [38]. While, whether an increase in p62 is related to improvement of cell aging is unclear. A recent study showed that p62 protein increased stability and longevity of nematode protein [30]. Therefore, we knocked down p62 protein expression in FGSCs, and further tested the anti-aging effect of SPD on H_2_O_2_-induced FGSCs. We found that SPD ameliorated cellular senescence of FGSCs through p62. These results suggest that SPD activates autophagy of FGSCs by inhibiting the PI3K/AKT/mTOR pathway. Additionally, SPD might achieve its anti-aging effect by promoting expression of p62 protein, which is located downstream of autophagic flow. Therefore, upregulation of p62 expression is indispensable for SPD ameliorating senescence of FGSCs caused by damage from oxidative stress.

**Fig. 7:**
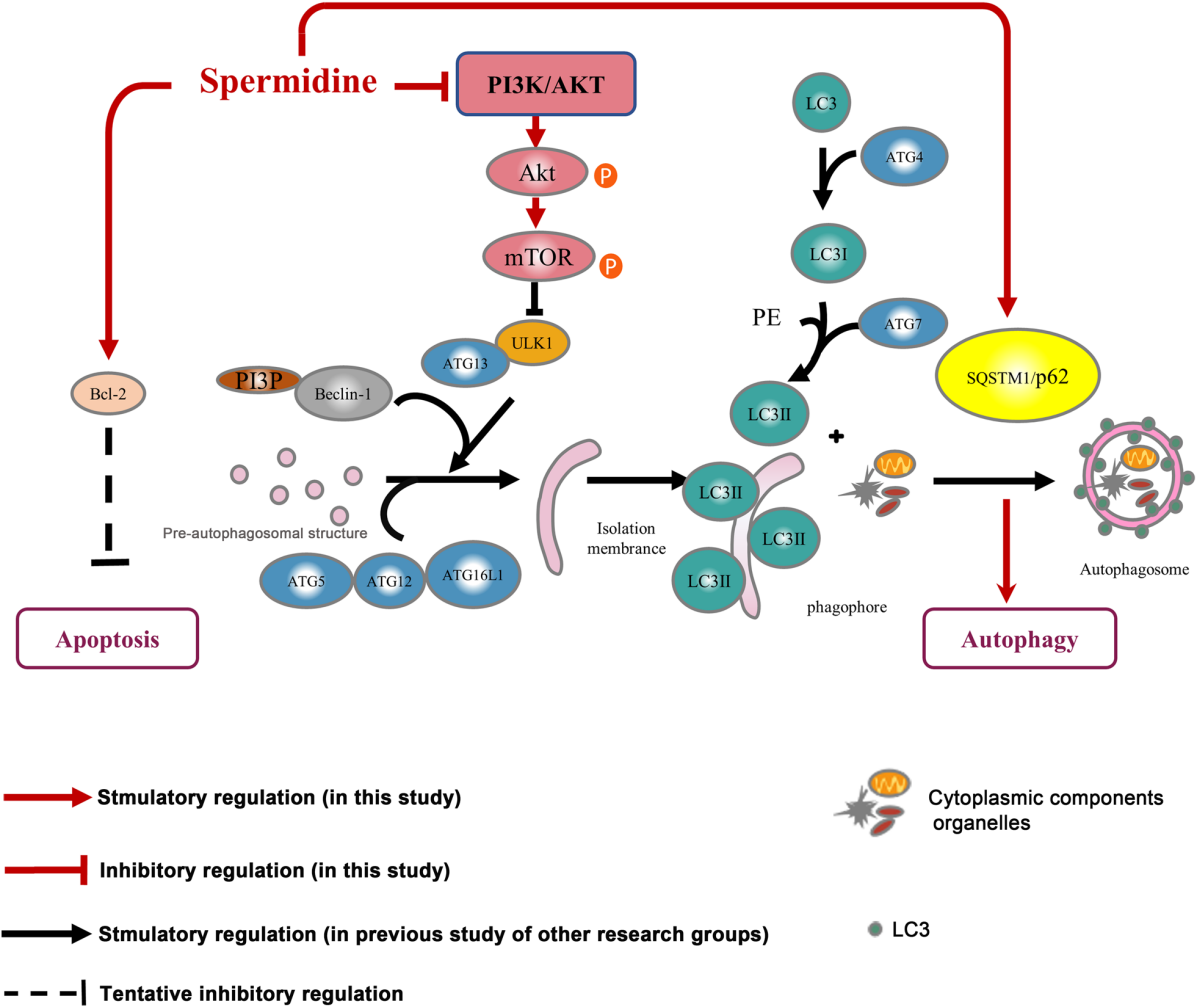
Proposed model of the regulatory mechanism of SPD-induced cytoprotective autophagy. SPD initiated autophagy via PI3K/AKT/mTOR signaling pathway. As a downstream target of the AKT/mTOR pathway, p62 may be a target protein for anti-aging. SPD maybe upregulate *Bcl-2* mRNA and inhibit apoptosis

Autophagy, similar to apoptosis and senescence, is an important biological phenomenon [39]. Research has focused on the idea that autophagy, apoptosis, and aging are connected in vivo to maintain the process of life in recent years, especially in the field of medical biology. Autophagy and autophagic flux generally decrease with age, and decreased autophagy is known to stimulate senescence of the body. Active autophagy can delay cellular senescence to extend the lifespan [40]. However, how impaired autophagy leads to cellular senescence needs to be further investigated. Occurrence of autophagy is a continuous process, also known as autophagic flux, including occurrence of autophagic precursors, formation of autophagosomes, transport of autophagosomes to lysosomes to form autophagic lysosomes, and degradation and recycling of substances in autophagic lysosomes. Recently, an increase in autophagosomes was found to not reflect the level of autophagy in nature, but only reflect induction of autophagy and inhibition of autophagosomes [41, 42]. With regard to autophagic flux, the number of autophagosomes is affected by formation and clearance. Therefore, accurately and comprehensively evaluating autophagy not only to detect autophagosomes, but also to dynamically observe the whole process of autophagic flux, is necessary. Our study showed that SPD-induced autophagy played a cytoprotective role in FGSCs by significantly increasing the number and viability of cells. We also analyzed and compared differentially expressed genes between the SPD treatment group and the control group by RNA-seq. GO and KEGG analysis showed a significant association of differentially expressed genes with the PI3K/AKT signaling pathway. AKT is a key signal transduction molecule in the PI3K/AKT signaling pathway, and its activation depends on PI3K. Phosphorylated AKT (p-AKT) is often used as an index to measure the activity of PI3K. mTOR is a specific downstream substrate of the AKT signaling pathway, which belongs to the family of PI3K-associated protein kinases. An increase in PI3K/AKT activity can activate mTOR by phosphorylation and mTOR is related to cell apoptosis, autophagy, and proliferation. Moreover, mTOR signaling can control lifespan and influences aging-related processes, such as cellular senescence [39]. Therefore, we further investigated the effect of SPD on the phosphorylation levels of AKT and mTOR. We found that SPD initiated autophagy by inhibiting phosphorylated expression of mTOR receptor. Our results suggest that SPD not only induces autophagy, but also reverses progress of aging of FGSCs caused by oxidative stress. Simultaneously, SPD promoted proliferation of FGSCs. Cellular proliferation and survival may have been related to cytoprotective autophagy. Additionally, upregulation of *Bcl-2* mRNA was induced by SPD (results of RNA-seq) (Fig. 7). However, owing to the complexity of the intracellular regulation mechanism, it may involve interaction of multiple pathways. This issue needs to be investigated by further studies.

## Conclusion

Our study further investigated the anti-aging mechanism of mTOR receptor, and p62, which is a downstream target of the AKT/mTOR pathway, may be a target protein for anti-aging (Fig. 7). We further expanded previous understanding of the function of p62 protein. Accumulation of p62 may not necessarily block degradation of autophagic lysosomes and inhibit autophagic flux, but may also have other biological effects, such as anti-aging. The specific regulatory mechanism for how inhibition of mTOR receptor phosphorylation affects p62 expression needs to be further investigated. Furthermore, we will continue to explore more the relationship between autophagy and cell senescence. Our research on SPD improving aging of FGSCs caused by H_2_O_2_ will provide a good direction for searching for clinical therapeutic drugs to maintain intracellular homeostasis in the future. Because of the unique characteristics of SPD (naturally non-toxic, drug efficacy with tissue specificity, and a dietary nutrient) it is likely to be used as an anti-aging drug in the future.

## Methods

### Chemical compound

Spermidine (05292), 3-MA (M9281), and CQ (C6628) were purchased from Sigma-Aldrich. SPD was in liquid form, which was diluted directly with culture medium to prepare different concentrations.

### Animals

Five-day-old mice were purchased from SLRC Laboratory (Shanghai, China). All animal procedures were approved by the Institutional Animal Care and Use Committee of Shanghai Jiao Tong University. The procedures were also performed in accordance with the National Research Council Guide for the Care and Use of Laboratory Animals.

### Cultures of FGSCs

In this study, we used the FGSC line, which was described in previous reports [8, 11, 12]. In brief, the FGSCs were cultured in minimum essential medium alpha (Invitrogen, Carlsbad, CA, USA), which was supplemented with 10% fetal bovine serum (Life Technologies, Carlsbad, CA, USA), 2 mM l-glutamine (Amresco, Radnor, PA, USA), 30 μg/mL pyruvate (Amresco), 1 mM nonessential amino acids (Invitrogen Life Sciences, CA, USA), 6 mg/mL penicillin (Amresco), 10 ng/mL mouse basic fibroblast growth factor (PeproTech, London, UK), 10 ng/mL mouse glial cell line-derived neurotrophic factor (PeproTech, NJ, USA), 20 ng/mL mouse epidermal growth factor (PeproTech), 10 ng/mL mouse leukemia inhibitory factor (Santa Cruz Biotechnology), and 50 mM β-mercaptoethanol (Sigma Chemical Co., St. Louis, MO, USA). SIM-6-thiogunaniaoualiain cells (ATCC, Manassas, VA, USA) served as the feeder to culture FGSCs, and were passaged every 4 days. FGSCs were identified by RT-PCR and immunofluorescence (Additional file 1: Figure S2). We used a hemocytometer to count the cell number per well.

### Infection of shRNA lentiviruses

FGSCs were split 1 day before infection to 50% confluence and infected with lentiviruses expressing shRNA-p62 for 24 h, according to the manufacturer’s protocol. To obtain the best sequence for p62 knockdown, three independent shRNA sequences targeting p62 were designed (Additional file 1: Table S1). Western blotting and immunofluorescence staining were performed to validate knockdown efficiency (Additional file 1: Fig. S3A, B). Finally, shRNA-p62 (5ʹ-GCTGAAACATGGACACTTTGG-3ʹ), which was cloned in the lentiviral vector of pGMLV-hU6-MCS-CMV-ZsGreen1-PGK-Puro-WPRE with a puromycin resistant region (Genmeditech, Shanghai, China), was selected. The infected cell lines were selected by puromycin (5 µg/mL) for 3 to 4 days to obtain stable knockdown of p62.

### mRFP-GFP-LC3 lentivirus transduction

A lentivirus encoding mRFP-GFP-LC3 was purchased from HanBio Inc. (Shanghai, China). Before transduction, equal numbers of FGSCs were plated in 24-well plates. When the cells reached approximately 50% confluence, they were infected with mRFP-GFP-LC3 lentivirus particles at a Multiplicity of Infection (MOI) of 1000 for 16 h. To obtain stable clones, the infected cell lines were selected by puromycin (5 µg/mL) for 3 to 4 days. Once established, these stable clonal cells were subjected to SPD or CQ treatment. Following treatment, 4% paraformaldehyde was used to fix the cells, and a fluorescence microscope was used to observe the number of GFP-positive and mRFP-positive dots.

### CCK-8 assay

When the cells reached 80% confluence, they were treated with different concentrations of SPD (0, 50, 100, and 200 μM) for 24 h. A volume of 100 mL CCK8, which was diluted 1:100 in cell culture medium, was then added to each well and incubated for 1 h at 37 °C. The optical density value was detected at 450 nm using a microplate spectrophotometer (Bio-Tek Instruments, Thermo Fisher Scientific, Winooski, VT, USA).

### Western blotting

Cells were lysed in radioimmunoprecipitation assay buffer (Yeasen, Co., Ltd., Shanghai, China), and protein was collected by centrifugation (4 °C at 12,000×*g* for 10 min). Protein concentrations were determined by the bicinchoninic acid protein assay kit (Yeasen, Co., Ltd.). Protein was separated by 15% sodium dodecyl sulfate polyacrylamide gel electrophoresis (SDS-PAGE) and subsequently transferred onto polyvinylidene fluoride (PVDF) membranes. Membranes were blocked with 6% non-fat milk in Tris-buffered saline Tween 20 for 1.5 h with gentle shaking at room temperature. Membranes were incubated at 4 °C overnight with the following primary antibodies: anti-β-tubulin (Santa Cruz Biotechnology), anti-LC3B (Cell Signaling Technology, Danvers, MA, USA), anti-p62 (Cell Signaling Technology), anti-AKT (Cell Signaling Technology), anti-phospho-AKT (Ser473; Cell Signaling Technology), mTOR (Abcam Inc., Cambridge, MA, USA), anti-phospho-mTOR (Ser2448; Abcam Inc.), anti-CDKN2A/p16INK4α (Abcam Inc.), and anti-p53 (Cell Signaling Technology). The membranes were washed three times with Tris-buffered saline Tween 20 for 15 min and incubated for 1 h with secondary antibodies (Proteintech, Rosemont, IL, USA) at room temperature. Protein brands were visualized using ECL Plus reagent (Beyotime, Shanghai, China) and scanned using a Tanon 4600SF (Tanon, Shanghai, China). The density of protein bands was quantified with Image-Pro Plus 6.0 software. The ratio of target protein to β-tubulin, which reflects changes in expression levels, was calculated.

### Immunofluorescence staining

FGSCs were fixed with 4% paraformaldehyde and then blocked with a solution containing 25% bovine serum albumin and 0.5% Triton-X 100. After incubation overnight at 4 °C with primary antibodies against LC3B, MVH or OCT4, cells were washed with phosphate-buffered saline and then further incubated for 90 min away from light with an appropriate fluorescence-conjugated secondary antibody. Nuclei were stained with 4ʹ,6-diamidino-2-phenylindole, dihydrochloride. Finally, images of the cells were acquired by a fluorescence microscope.

### RNA isolation and sequencing

Total RNA was extracted from FGSCs using Trizol reagent (Life Technologies). The corresponding cDNA libraries were constructed using the VAHTSTM mRNA-seq v2 Library Prep Kit for Illumina1 (Vanzyme, Co., Ltd., Shanghai, China), and sequencing libraries were created according to the manufacturer’s protocol. The library quality was determined using a Bioanalyzer 2100 (Agilent, Santa Clara, CA, USA) and sequenced with an Illumina Hiseq2500 platform (Illumina, San Diego, CA, USA). RNA-seq reads were aligned to reference data that were downloaded from UCSC (version hg19). The Reads Per Kilobase Million (RPKM) method was used to normalize reads that were exclusively mapped to a gene to quantify the transcript levels. The quality of RNA-seq data was evaluated using FastQC. Raw sequencing data have been submitted to the NCBI Sequence Read Archive under Accession number GSE164387.

### GO and KEGG pathway analysis

GO analysis was performed to identify the biochemical process of unique genes of differentially expressed mRNAs. KEGG pathway analysis was applied to identify significant pathways of the differentially expressed mRNAs. We uploaded the data of differentially expressed mRNAs to DAVID (http://david.abcc.ncifcrf.gov/home.jsp). We obtained the enrichment results. Fisher’s exact test was used to identify significant results and the false discovery rate was applied to correct the P values (P < 0.05; fold change > 1.5).

### qRT-PCR and RT-PCR

Approximately 1000 ng of RNA was reverse transcribed into cDNA. We then used an ABI 7500 Real-Time PCR system (Applied Biosystems, Foster City, CA, USA) to detect mRNA expression levels. The qRT-PCR conditions were as follows: 95 °C for 30 s, followed by 40 cycles of 95 °C for 5 s and 60 °C for 34 s, and then 95 °C for 15 s, 60 °C for 60 s, and 95 °C for 15 s. The RT-PCR conditions consisted of initial denaturing at 95 °C for 5 min, followed by 30 cycles at 95 °C for 30 s, annealing at 55 °C for 30 s, and extension at 72 °C for 30 s, and a final extension at 72 °C for 10 min. PCR products were examined on an agarose gel stained with ethidium bromide. All qRT-PCR and RT-PCR experiments were repeated three times, and relative mRNA expression was then calculated using the ΔΔCt method [43] with *Gapdh* as the internal control. All primers used for qRT-PCR and RT-PCR are listed in Additional file 1: Tables S2, S3 (Generay Biotech Co., Ltd., Shanghai, China).

### Statistical analysis

Experiments were performed at least three times and data are expressed as mean ± SEM. One-way analysis of variance or the two-tailed Student’s t-test was used to analyze differences among different groups. Statistical analysis was performed with GraphPad Prism 6.0 software (San Diego, CA, USA) P < 0.05 was considered statistically significant.

## Supplementary Information

**Additional file 1: Table S1.** p62 shRNA sequences. **Table S2.** Sequence of primers used in this study. **Table S3.** Sequence of primers used in RT-PCR. **Figure S1.** Quality control of RNA-seq. **Figure S2.** Characteristics of FGSCs. **Figure S3.** Verification of p62-knockdown efficiency.

## Data Availability

All data generated or analyzed during this study are included in this published article.

## Abbreviations

SPD: Spermidine
FGSCs: Female germline stem cells
MAP1LC3B/LC3B: Microtubule-associated protein 1 light chain 3 beta
SQSTM1/p62: Sequestosome-1
CQ: Chloroquine
3-MA: 3-Methyladenine
p16: Cyclin dependent kinase inhibitor 2A
p53: Tumor protein 53
BCL2: B cell leukemia/lymphoma 2
PI3K: Phosphoinositide 3-kinase
AKT: AKT serine/threonine kinase
mTOR: Mechanistic target of rapamycin kinase
H_2_O_2_: Hydrogen peroxide
SA-GLB1/β-gal: Senescence-associated galactosidase beta 1
KD: Knockdown
qPCR: Quantitative PCR
WB: Western blotting
