# Supplementary material for: Spermidine induces cytoprotective autophagy of female germline stem cells in vitro and ameliorates aging caused by oxidative stress through upregulated sequestosome-1/p62 expression
Source: Cell Biosci. 2021 Jun 7;11:107. doi: 10.1186/s13578-021-00614-4 (PMC8186080; doi:10.1186/s13578-021-00614-4)
Supplement: Supplementary file 1 — Additional file 1: Table S1. p62 shRNA sequences. Table S2. Sequence of primers used in this study. Table S3. Sequence of primers used in RT-PCR. Figure S1. Quality control of RNA-seq. Figure S2. Characteristics of FGSCs. Figure S3. Verification of p62-knockdown efficiency. [file 13578_2021_614_MOESM1_ESM.pdf]

Additional file 1

Table S1. p62 shRNA sequences

No.	Sequences 5' to 3'
p62 shRNA1	GCTGAAACATGGACACTTTGG
p62 shRNA2	GCTCCTACAGACCAAGAATTA
p62 shRNA3	GCAGTGTGTGCCCAGACTACG
Con shRNA	TTCTCCGAACGTGTCACGT

Table S2. Sequence of primers used in this study

Gene	PCR primer [a.]	Product size
	qRT-PCR	
Akt3	F: TGGGTTCAGAAGAGGGGAGAA	122bp
	R: AGGGGATAAGGTAAGTCCACATC	
Tcl1b1	F: CACTTCCAGTCTACCTGGTCT	132bp
	R: GGACAGTTACATGGGTTCTCCT	
Col4a4	F: GCCTGGTGTCGGGATCAAAG	211bp
	R: AGCTGGAGTCAACAAAATGCC	
Lpar3	F: CAAGCGCATGGACTTTTTCTAC	211bp
	R: GAAATCCGCAGCAGCTAAGTT	
Gapdh	F: TGGATTTGGACGCATTGGTC	211bp
	R: TTTGCACTGGTACGTGTTGAT	

a. F, forward; R, reverse.

Table S3 Sequence of primers used in RT-PCR

Gene	PCR primer [a.]	Product size
Mvh	F: GGAAACCAGCAGCAAGTGAT<br>R: TGGAGTCCTCATCCTCTGG	213bp
Oct4	F: CTCGAACCACATCCTTCTCT<br>R: GGCGTTCTCTTTGGAAAGGTGTTC	334bp
Dazl	F: GTGTGTCGAAGGGCTATGGAT<br>R: ACAGGCAGCTGATATCCAGTG	328bp
Stella	F: CCCAATGAAGGACCCTGAAAC<br>R: AATGGCTCACTGTCCCGTTCA	354bp
Gapdh	F: GTCCCGTAGACAAAATGGTGA<br>R: TGCATTGCTGACAATCTTGAG	458bp

b. F, forward; R, reverse.

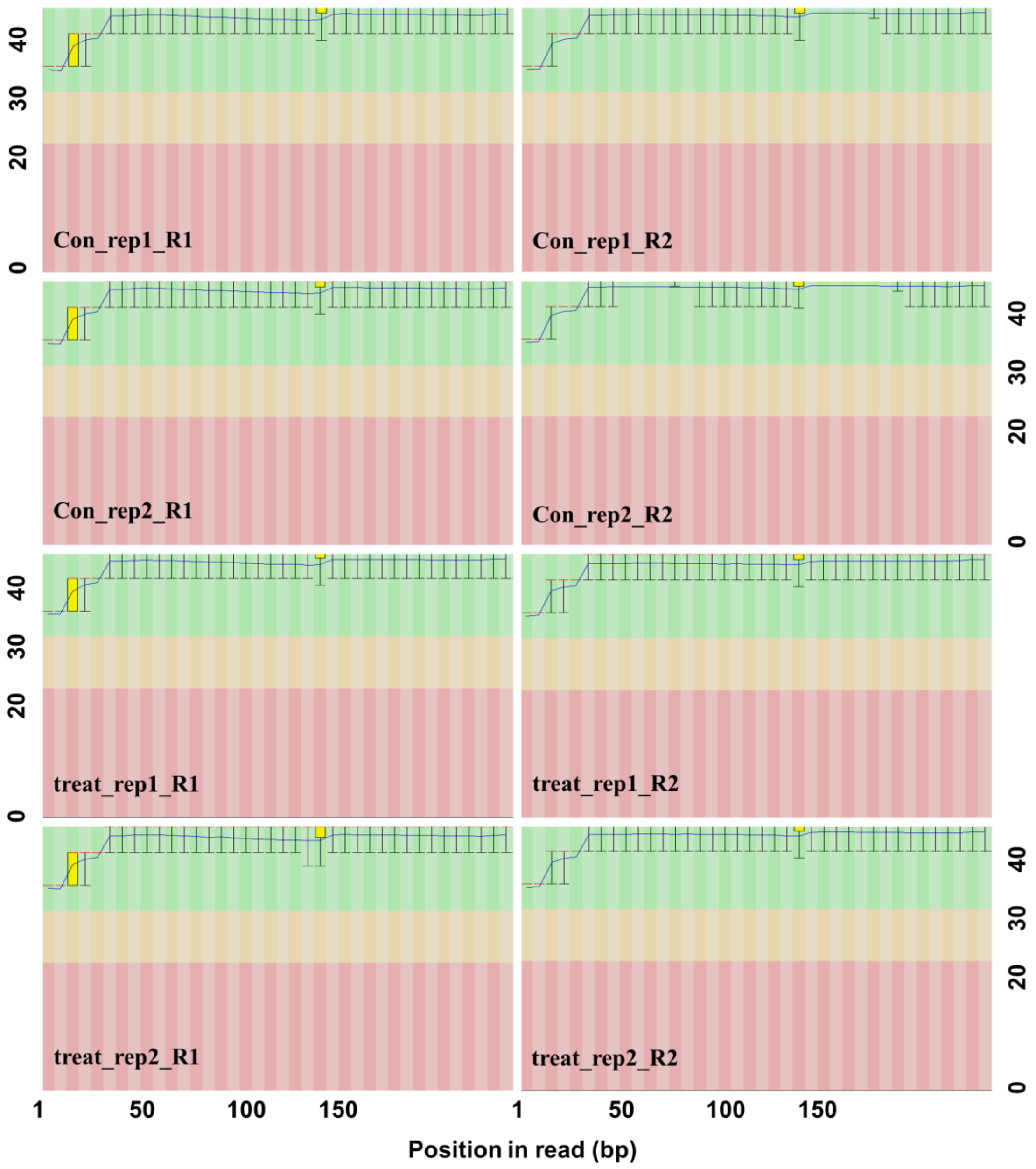

Figure S1. Quality control of RNA-seq. FastQC data showing the position-specific sequencing quality in each replicated group. Data filtering criterion was a Q-score > 10 (error rate < 10%). Con: untreated FGSCs. treat: SPD-treated FGSCs.

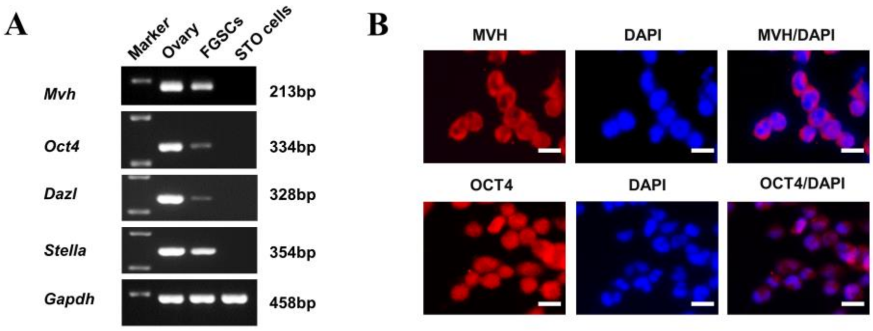

Figure S2. Characteristics of FGSCs. (A) The mRNA expression of FGSCs markers were detected by RT–PCR. STO is negative control. Ovary from adult mice was used as a positive control. Glyceraldehyde 3-phosphate dehydrogenase (*Gapdh*) expression was used as an internal control. *Mvh*, 213 bp; *Oct4*, 334 bp; *Dazl*, 328 bp; *Stella*, 354 bp; *Gapdh*,458 bp. **(B)** Immunofluorescence staining of MVH and OCT4 in FGSCs (scale bars: 20 μm).

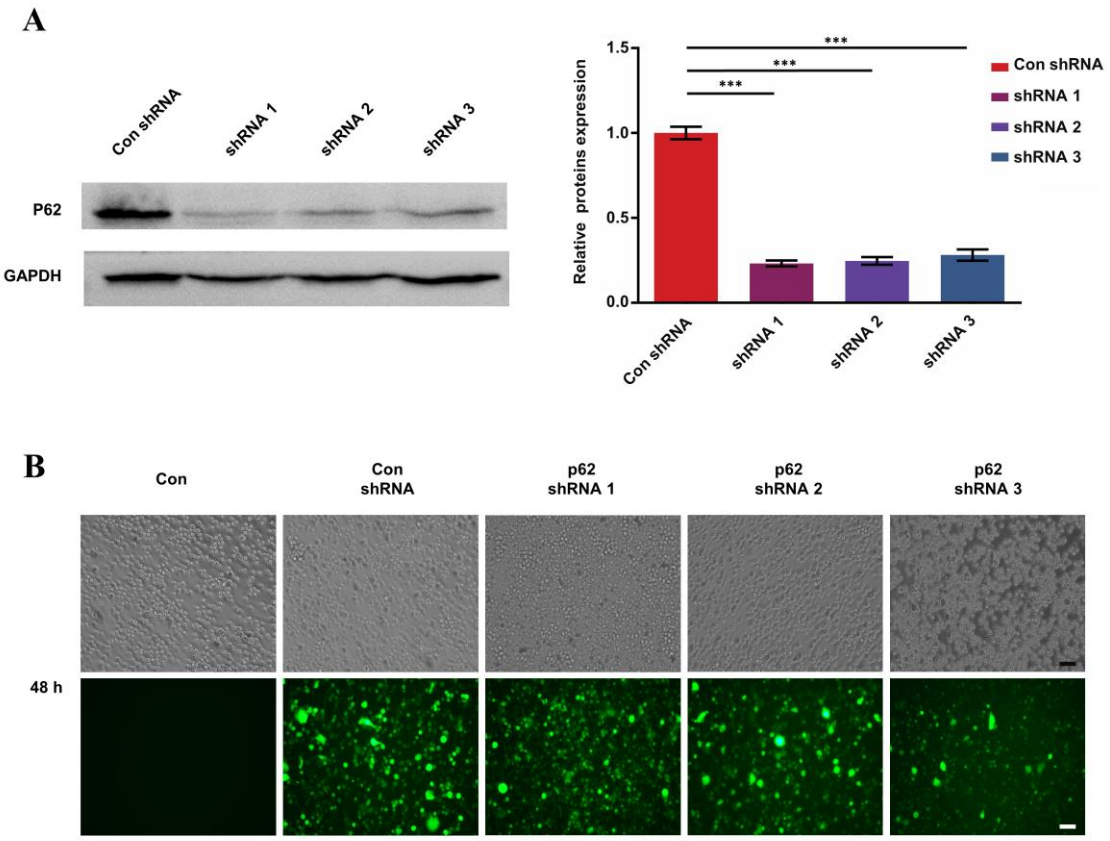

Figure S3. Verification of p62-knockdown efficiency. (A) Western blotting was performed to validate knockdown efficiency of three shRNA lentiviruses. All data are expressed as the mean ± SEM of values from experiments performed at least in triplicate. ***P < 0.001 compared with controls. **(B)** Fluorescence microscopy was used to determine the infection efficiency of three shRNA lentiviruses. Con: control.
